# Colon Effects of 7-Day Whole-Body Inhalation Exposure of Fischer 344 Rats to Emissions from First- and Second-Generation Biodiesel Fuels with and Without a Particle Filter

**DOI:** 10.3390/ijms27219724

**Published:** 2026-10-29

**Authors:** Jacek Wilczak, Katarzyna Dziendzikowska, Joanna Gromadzka-Ostrowska, Michał Oczkowski, Rafał Sapierzyński, Malwina Czerwińska, Anna Lankoff

**Affiliations:** 1 Department of Basic Sciences, Faculty of Veterinary Medicine, Warsaw University of Life Sciences, Nowoursynowska 159, 02-776 Warsaw, Poland; 2 Department of Dietetics, Faculty of Health Sciences, Warsaw University of Life Sciences, Nowoursynowska 159, 02-776 Warsaw, Poland; katarzyna_dziendzikowska@sggw.edu.pl (K.D.); joanna_gromadzka-ostrowska@sggw.edu.pl (J.G.-O.); michal_oczkowski@sggw.edu.pl (M.O.); 3 Department of Pathology and Veterinary Diagnostics, Faculty of Veterinary Medicine, Warsaw University of Life Sciences, Nowoursynowska 159, 02-776 Warsaw, Poland; rafal_sapierzynski@sggw.edu.pl; 4 Centre for Radiobiology and Biological Dosimetry, Institute of Nuclear Chemistry and Technology, Dorodna 16, 03-195 Warsaw, Poland; m.czerwinska@ichtj.waw.pl (M.C.); a.lankoff@ichtj.waw.pl (A.L.); 5 Department of Medical Biology, Institute of Biology, Jan Kochanowski University, Uniwersytecka 7, 25-406 Kielce, Poland

**Keywords:** first- and second-generation biodiesel fuels, colon, lipid peroxidation, oxidative stress, antioxidant defence, inflammatory response

## Abstract

Biodiesel is projected to reduce greenhouse gas and particulate matter emissions relative to petrodiesel, but biodiesel engine emissions (DEEs) contain higher levels of nitrogen oxides, nano-sized particles, and specific oxygenated volatile organic compounds and aromatics that may increase health risks. Most research on DEE health effects has focused on respiratory, cardiovascular, neurodegenerative, and reproductive outcomes, leaving a considerable knowledge gap regarding gastrointestinal effects. We therefore examined and compared the colon effects of DEE produced by combustion of first-generation B7 biofuel (7% *v*/*v* fatty acid methyl esters, FAMEs) or second-generation SHB20 biofuel (7% *v*/*v* FAME plus 13% *v*/*v* hydrotreated vegetable oils, HVOs), with or without a diesel particle filter (DPF). Male Fischer 344 rats were exposed to DEE for 7 consecutive days (6 h/day; seven exposure sessions, 42 h in total) in whole-body inhalation chambers, at measured particulate matter concentrations of 2.19 and 2.13 mg/m^3^ without DPF and 0.17 and 0.19 mg/m^3^ with DPF for B7 and SHB20, respectively. Whole-body DEE exposure induced lipid peroxidation, oxidative-stress-associated and redox-related changes, mobilisation of the antioxidant defence system, and an inflammatory-associated response in colon tissue. These colon effects were blend-specific and only partly and endpoint-dependently mitigated by DPF use, with SHB20 DEE without DPF producing the most pronounced responses.

## 1. Introduction

In 2018, the European Union adopted the second iteration of the Renewable Energy Directive II (RED II) for the period 2021–2030, also known as the biofuel directive, establishing binding requirements for renewable energy use. This directive sets biofuel consumption targets, specifying that by 2030, renewable energy sources must constitute at least 32.5% of the total energy mix, including at least 14% in the transport sector [1]. Although the United States does not have a national target for biofuel consumption, it has implemented the Renewable Fuel Standard (RFS) programme, administered by the Environmental Protection Agency (EPA), which mandates a steady increase in biofuel use in the transportation sector. According to the EPA final rule for 2023–2025, the total renewable fuel share is expected to reach 13.05% by 2025 [2]. Apart from the European Union and the United States, more than 60 countries have launched programmes that require or promote the blending of biofuels [3]. Consequently, in high-income countries, biofuel consumption in the transport sector is projected to increase by 1% to 6% per year, while in middle-income countries, it is expected to grow by 1.7% annually over the next decade. This means that people’s exposure to biofuel combustion will steadily increase as global production and use rise.

One of the most commonly used types of biofuels today is biodiesel, which is classified into four generations based on feedstock origin and production technology [4]. First-generation biodiesel consists of fatty acid methyl esters (FAMEs) produced from conventional food crops or animal fats via transesterification. The second-generation biodiesel, known as hydrogenation-derived renewable diesel (HDRD) or hydrotreated vegetable oil (HVO), is produced from non-food sources, such as used cooking oils and animal fats, via hydrotreating. In contrast, the third-generation algae-based biofuels and fourth-generation biofuels derived from algal metabolic engineering remain under development and are not yet widely implemented [5]. Whilst there are potential environmental benefits of introducing biodiesel fuels, such as a decrease in greenhouse gas, hydrocarbons and particulate matter emissions during combustion, substantial evidence indicates that these diesel exhaust emissions (DEEs) may contain higher levels of nitrogen oxides and nano-sized particles, more particle-bound specific oxygenated volatile organic compounds and aromatics [6], which may increase the risk of adverse health effects [7].

The majority of studies investigating the health effects of DEE have focused on the respiratory and cardiovascular outcomes [8,9,10,11]. In contrast, only a few studies reported associations between exposure to DEE and neurodegenerative [12,13,14] and reproductive effects [15,16]. However, a significant knowledge gap exists regarding the potential impact of DEE exposure on the gastrointestinal tract. Although a limited number of studies have shown that gut microbiome composition and metabolic profiles are altered after DEE [17,18], the direct effects of these emissions on the gastrointestinal tract remain unknown.

In response to this lack of knowledge, the current study was designed to examine and compare the effects of DEE generated by the combustion of first-generation B7 biofuel and second-generation SHB20 biofuel, with and without a diesel particle filter (DPF), on the colon. The first-generation B7 biofuel contained 7% (*v*/*v*) FAME, whereas the second-generation SHB20 biofuel consisted of 7% (*v*/*v*) FAME and 13% (*v*/*v*) HVO. Colon toxicity was evaluated in Fischer 344 rats following exposure to DEE for 7 consecutive days (6 h/day) in whole-body inhalation chambers. The potential toxicity of DEE in rat colon tissue was assessed by measuring lipid peroxidation, mobilisation of the antioxidant defence system, inflammatory responses, and histopathological alterations. Furthermore, an extensive chemical analysis of DEE was conducted, covering gaseous combustion products and particulate matter. This study provides the first evidence that whole-body exposure of rats to DEE, produced by the combustion of the first-generation B7 biofuel or the second-generation SHB20 biofuel with and without DPF, is associated with the induction of lipid peroxidation, oxidative stress, mobilisation of the antioxidant defence system and an inflammatory response in the colon tissue.

## 2. Results

### 2.1. The Characteristics of Selected Substances in the Test Chambers

The detailed methodology for analysing air composition within the inhalation chambers, together with the detailed results, has been described in our previous studies [19,20,21,22]. Briefly, no differences in concentrations of carbon monoxide (CO), carbon dioxide (CO_2_), nitrogen oxides (NOx), and particulate matter (PM) were observed between exhaust emissions generated from B7 and SHB20 biofuels (Supplementary Table S1). However, B7 exhaust emissions contained ~1.5-fold higher concentrations of unburned hydrocarbons (HCs) than SHB20 exhaust emissions. The use of DPF significantly reduced CO and PM concentrations in both exhaust types.

The DEPs were collected from the main diesel exhaust without a diesel particulate filter on PTFE-coated glass fibre filters (70 mm; Pallflex, Emfab filters, TX40HI20WW). The filters were weighed before and after sampling to determine the total particulate matter collected. The filters were pooled to obtain a sufficient amount of DEPs for physicochemical analyses. The DEPs were scraped from the filters and extracted with dichloromethane. The amount of DEPs collected from the main diesel exhaust with a diesel particulate filter was insufficient to perform physicochemical analyses; therefore, these analyses were only conducted on samples collected from biofuel combustion without a DPF.

Both B7 and SHB20 exhaust emissions, with and without DPF, were further analysed for the presence of 17 polycyclic aromatic hydrocarbon (PAH) compounds (Supplementary Figure S1). Without DPF, the total PAH concentration in B7 exhaust emissions (38,382.31 pg/m^3^) was ~2.5-fold higher than in SHB20 exhaust emissions (15,129.05 pg/m^3^). Pyrene, fluoranthene, phenanthrene, and chrysene were the most abundant PAHs in emissions without DPF. The application of DPF reduced total PAH concentrations by approximately 1000-fold in B7 exhaust emissions (37.83 pg/m^3^) and 250-fold in SHB20 exhaust emissions (61.35 pg/m^3^). Under filtered conditions, chrysene, naphthalene, benzo(b)fluoranthene, benzo(a)anthracene, and benzo(k)fluoranthene were the predominant PAHs.

Comparative analysis of PM concentrations inside inhalation chambers showed similar PM levels in exhaust emissions generated from B7 and SHB20 biofuels without a DPF. The use of DPF reduced PM concentrations by approximately 13-fold in both exhaust types. However, detailed PM analysis revealed differences in particle composition between fuels, with higher concentrations of transition metals (iron, zinc, and copper) and the presence of silver in particles generated from SHB20 combustion (Supplementary Figure S2). Moreover, analysis of PAHs in organic extracts from particles generated during B7 and SHB20 combustion showed that total PAH concentrations were ~2.4-fold higher in B7-derived particles (165.78 ng/mg) than in SHB20-derived particles (69.93 ng/mg). Pyrene, fluoranthene, phenanthrene, and chrysene were the predominant PAHs in all samples.

### 2.2. Exposure to DEE from Combustion of the First-Generation B7 and the Second-Generation SHB20 Biofuels with and Without DPF Induces Lipid Peroxidation in the Colon of Fischer 344 Rats

**Figure 1:**
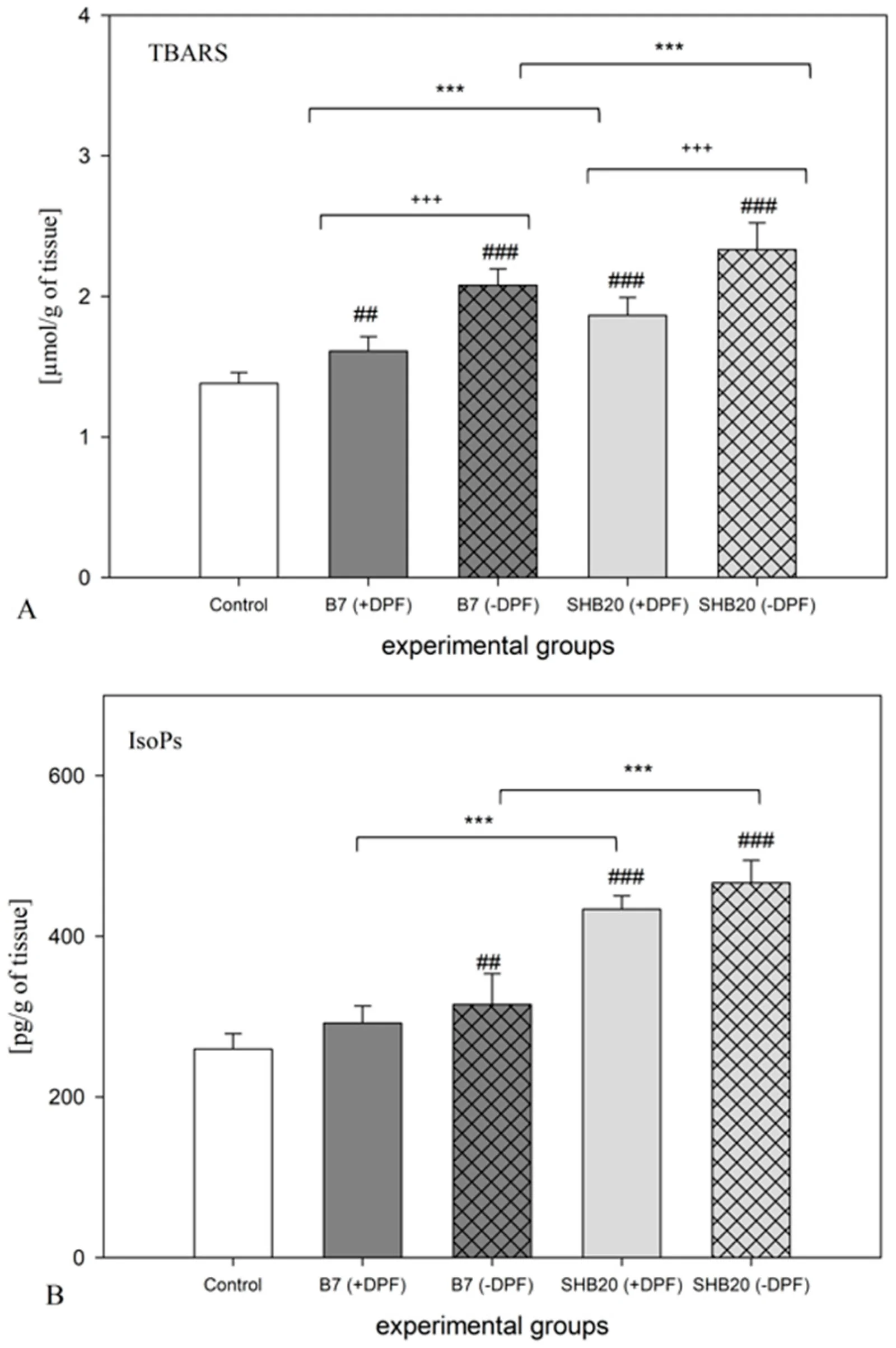
The concentration of thiobarbituric acid-reactive substances (TBARSs) [µmol/g of tissue] (**A**) and isoprostanes (ISOPs) [pg/g of tissue] (**B**) in the colon tissue of rats exposed to DEE from B7 and SHB20 biofuels with (+DPF) and without DPF treatment (-DPF) for 7 days. Data are expressed as mean ± SD; *n* = 7 per group. #, Statistically significant difference from the control group; ##, *p* < 0.01; ###, *p* < 0.001; one-way ANOVA with Dunnett’s post hoc test. *, Statistically significant difference between B7 and SHB20 DEE; ***, *p* < 0.001; +, statistically significant difference between treatment with and without DPF; +++, *p* < 0.001; two-way ANOVA (biofuel type × DPF treatment) with Tukey’s post hoc test.

One of the most widely used assays for measuring ROS-induced lipid peroxidation of polyunsaturated fatty acids is the TBARS assay. As illustrated in Figure 1A, TBARS concentrations in colon tissue were significantly increased in all groups of rats exposed to DEE generated from B7 and SHB20 biofuels, with and without DPF treatment [+17% in B7 (+DPF), +50% in B7 (-DPF), +35% in SHB20 (+DPF), and +69% in SHB20 (-DPF)], compared to the control group. When comparing fuel types, significantly higher TBARS concentrations were observed in the SHB20 (+DPF) and SHB20 (-DPF) groups (+16% and +13%, respectively) than in the corresponding B7 (+DPF) and B7 (-DPF) groups. Moreover, significantly higher TBARS concentrations were observed in rats exposed to non-filtered B7 and SHB20 emissions (+29% and +25%, respectively) compared to the corresponding DPF-treated groups.

In addition to TBARS analysis, the concentration of isoprostanes (ISOPs), reliable biomarkers of oxidative stress and lipid peroxidation, was determined in colon tissues. As presented in Figure 1B, ISOP concentrations were significantly increased in three groups exposed to B7 and SHB20 DEE, with and without DPF treatment [+21% in the B7 (-DPF), +67% in the SHB20 (+DPF), and +80% in the SHB20 (-DPF) groups], compared to the control group. When comparing B7 and SHB20 groups, significantly higher ISOP concentrations were observed in the SHB20 (+DPF) and SHB20 (-DPF) groups (+49% and +48%, respectively) than in the corresponding B7 (+DPF) and B7 (-DPF) groups. DPF treatment did not affect ISOP concentrations.

### 2.3. Exposure to DEE from Combustion of the First-Generation B7 and the Second-Generation SHB20 Biofuels with and Without DPF Changes Antioxidant Status in the Colon of Fischer 344 Rats

**Figure 2:**
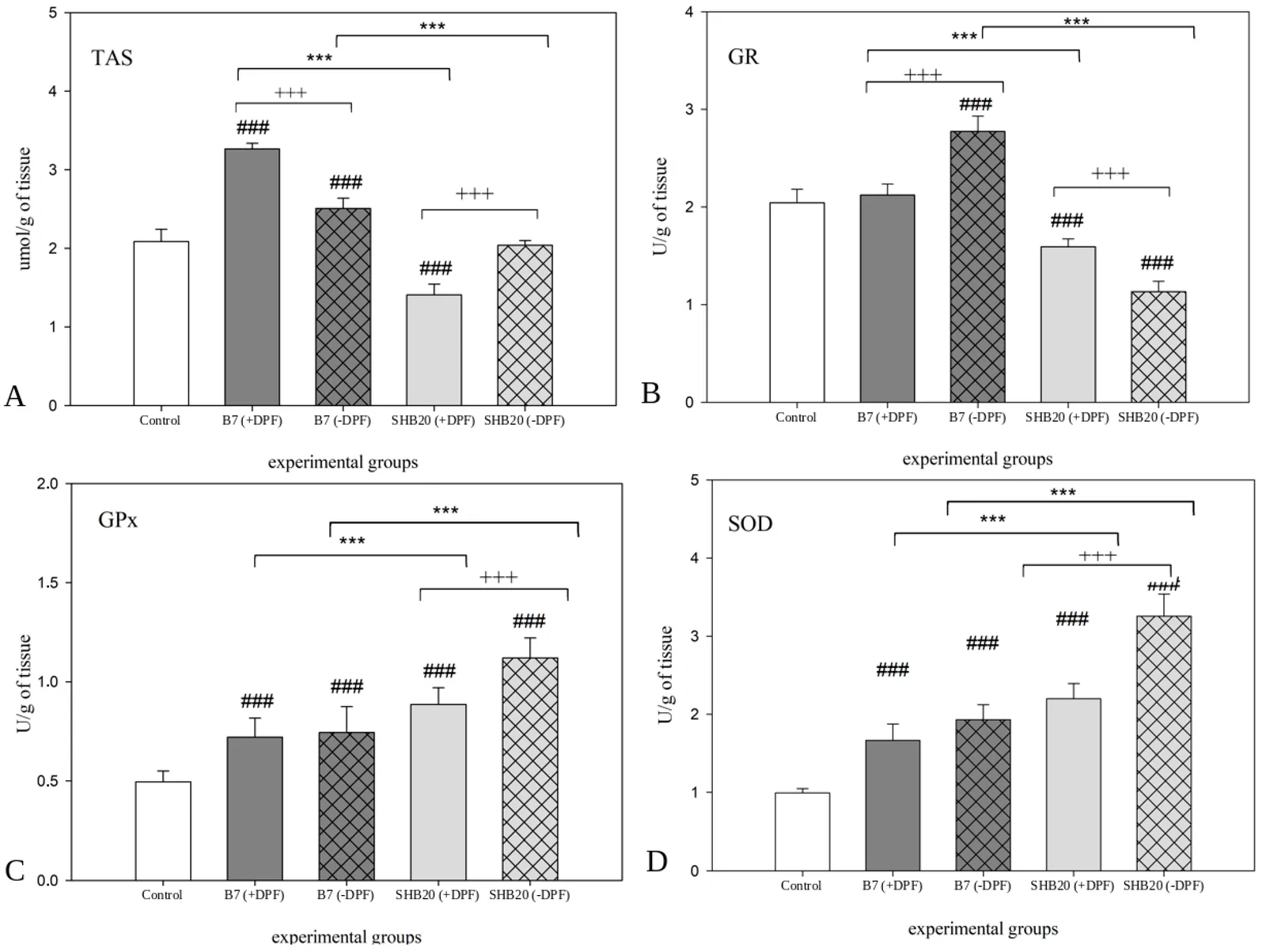
The total antioxidant status (TAS) [µmol/g of tissue] (**A**) and the activity of glutathione reductase (GR) [U/g of tissue] (**B**), glutathione peroxidase (GPx) [U/g of tissue] (**C**) and superoxide dismutase (SOD) [U/g of tissue] (**D**) in the colon of rats exposed to DEE from B7 and SHB20 biofuels with and without DPF treatment for 7 days. Data are expressed as mean ± SD; *n* = 7 per group. #, Statistically significant difference from the control group; ###, *p* < 0.001; one-way ANOVA with Dunnett’s post hoc test. *, Statistically significant difference between B7 and SHB20 DEE; ***, *p* < 0.001. +, Statistically significant difference between treatment with and without DPF; +++, *p* < 0.001; two-way ANOVA (biofuel type × DPF treatment) with Tukey’s post hoc test.

Total antioxidant status (TAS), one of the most widely used indicators of antioxidant defence in tissues, is presented in Figure 2A. TAS significantly increased in the B7 (+DPF) and B7 (-DPF) groups (+53% and +20%, respectively), whereas it markedly decreased by 33% in the SHB20 (+DPF) group, compared to the control group. When comparing fuel types, TAS levels were significantly higher in the B7 (+DPF) and B7 (-DPF) groups (+57% and +19%, respectively) than in the corresponding SHB20 (+DPF) and SHB20 (-DPF) groups. DPF treatment significantly increased TAS in the B7 (+DPF) group (+20%) compared to B7 (-DPF), while significantly decreasing TAS in the SHB20 (+DPF) group (−29%) compared to SHB20 (-DPF).

TAS is highly dependent on the primary enzymatic defence system, which includes glutathione reductase (GR), glutathione peroxidase (GPx), and superoxide dismutase (SOD). As shown in Figure 2B, GR activity significantly increased by 36% in the B7 (-DPF) group, whereas it markedly decreased by 22% and 45% in the SHB20 (+DPF) and SHB20 (-DPF) groups, respectively, compared to the control group. GR activity was significantly lower in the SHB20 (+DPF) and SHB20 (-DPF) groups (−25% and −59%, respectively) than in the corresponding B7 groups. In addition, GR activity was higher in the B7 (-DPF) group (+30%) than in B7 (+DPF), but lower in SHB20 (-DPF) (−29%) than in SHB20 (+DPF).

GPx activity (Figure 2C) significantly increased in all exposed groups [+46% in B7 (+DPF), +51% in B7 (-DPF), +80% in SHB20 (+DPF), and +126% in SHB20 (-DPF)], compared to the control group. When comparing fuel types, GPx activity was higher in the SHB20 (+DPF) and SHB20 (-DPF) groups than in the corresponding B7 groups (+22% and +34%, respectively). DPF treatment significantly decreased GPx activity by 26% in SHB20 (+DPF) compared with SHB20 (-DPF).

Similarly, SOD activity (Figure 2D) was significantly elevated in all groups exposed to B7 and SHB20 DEE [+67% in B7 (+DPF), +95% in B7 (-DPF), +120% in SHB20 (+DPF), and +228% in SHB20 (-DPF)], compared to the control group. SOD activity was significantly higher in the SHB20 (+DPF) and SHB20 (-DPF) groups than in the corresponding B7 groups (+32% and +68%, respectively). DPF treatment significantly decreased SOD activity by 45% in SHB20 (+DPF) compared to SHB20 (-DPF).

**Figure 3:**
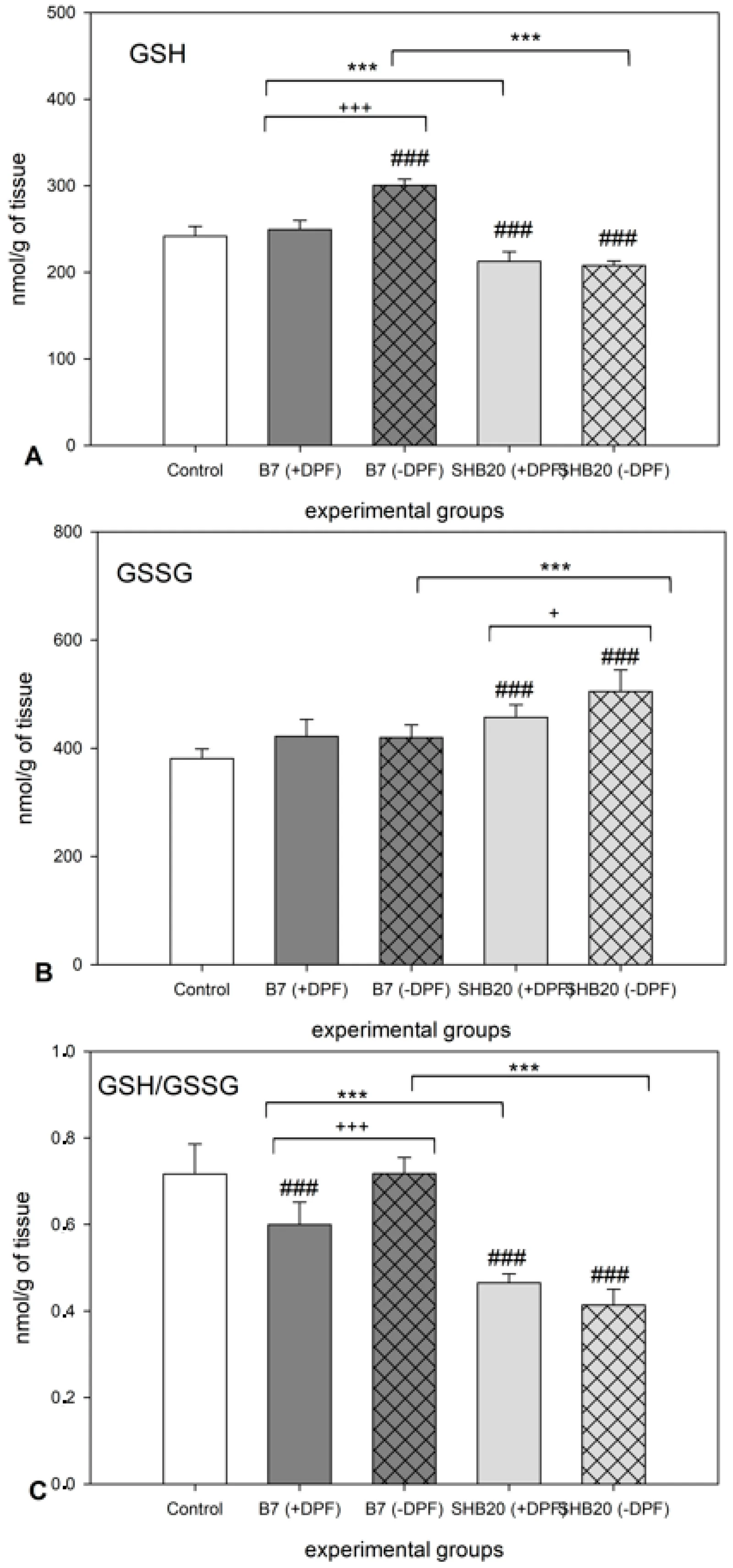
The concentration of reduced form of glutathione (GSH) [nmol/g of tissue] (**A**), oxidised form of glutathione (GSSG) [nmol/g of tissue] (**B**) and GSH-to-GSSG ratio (**C**) in the colon of rats exposed to DEE from B7 and SHB20 biofuels with and without DPF treatment for 7 days. Data are expressed as mean ± SD; *n* = 7 per group. #, Statistically significant difference from the control group; ###, *p* < 0.001; one-way ANOVA with Dunnett’s post hoc test. *, Statistically significant difference between B7 and SHB20 DEE; ***, *p* < 0.001. +, Statistically significant difference between treatment with and without DPF; +, *p* < 0.05; +++, *p* < 0.001; two-way ANOVA (biofuel type × DPF treatment) with Tukey’s post hoc test.

Apart from the enzymatic antioxidant defence parameters, non-enzymatic antioxidants, such as GSH and GSSG, also scavenge ROS and stabilise lipid membranes. As illustrated in Figure 3A, the concentration of GSH significantly increased by 24% in the B7 (-DPF) group of rats; however, it decreased by 12% and 14% in the SHB20 (+DPF) and SHB20 (-DPF) groups of rats, respectively, compared to the control group. When comparing B7 and SHB20 groups, significantly higher concentrations of GSH were observed in the B7 (+DPF) and B7 (-DPF) groups of rats (+15% and + 30%, respectively), compared to the corresponding SHB20 (+DPF) and SHB20 (-DPF) groups of rats.

When studying the effects of filtered versus non-filtered B7 and SHB20 DEE, a significantly higher GSH concentration was observed only in the B7 (-DPF) group (+17%) compared to the corresponding B7 (+DPF) group. As shown in Figure 3B, GSSG concentration was significantly increased in the SHB20 (+DPF) and SHB20 (-DPF) groups (+17% and +25%, respectively), compared to the control group. Moreover, GSSG concentration was higher in the SHB20 (-DPF) group (+17%) than in the corresponding B7 (-DPF) group, whereas DPF treatment reduced GSSG concentration by 10% in SHB20-exposed rats.

The GSH/GSSG ratio (Figure 3C) significantly decreased in the B7 (+DPF) (−17%), SHB20 (+DPF) (−36%), and SHB20 (-DPF) (−43%) groups, compared to the control group. Compared with the corresponding B7 groups, the GSH/GSSG ratio was lower in SHB20 (+DPF) and SHB20 (-DPF) rats (−28% and −42%, respectively). DPF treatment significantly increased the GSH/GSSG ratio by 20% in the B7 (+DPF) group compared with the B7 (-DPF) group.

### 2.4. Exposure to DEE from Combustion of the First-Generation B7 and the Second-Generation SHB20 Biofuels with and Without DPF Deregulates Pro/Anti-Inflammatory Cytokines in the Colon of Fischer 344 Rats

**Figure 4:**
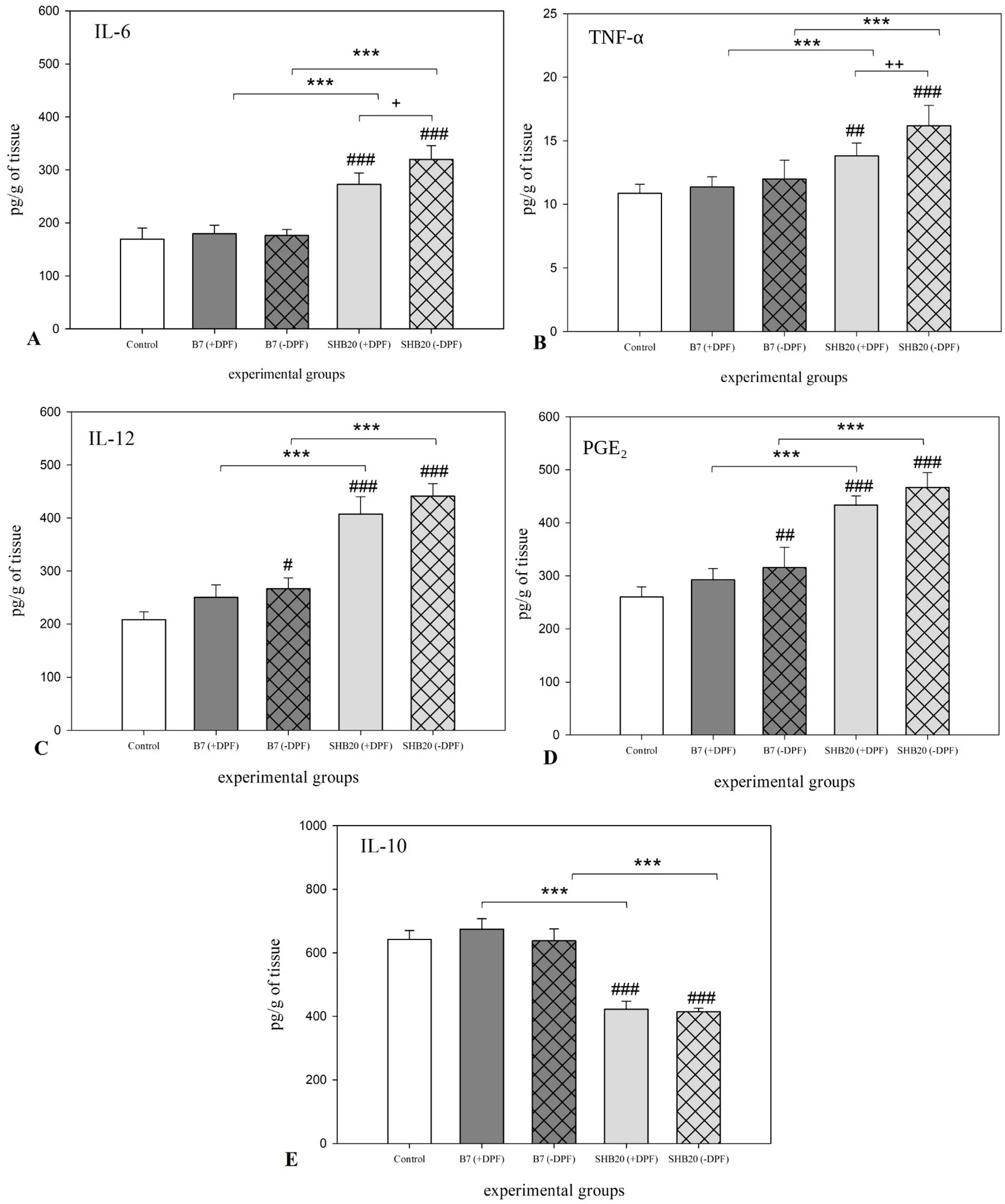
The concentration of interleukin 6 (IL-6) [pg/g of tissue] (**A**), tumour necrosis factor alpha (TNFα) [pg/g of tissue] (**B**), interleukin 12 (IL-12) [pg/g of tissue] (**C**), prostaglandin E2 (PGE2) [pg/g of tissue] (**D**) and interleukin 10 (IL-10) (**E**) in the colon of rats exposed to DEE from B7 and SHB20 biofuels with and without DPF treatment for 7 days. Data are expressed as mean ± SD; *n* = 7 per group. #, Statistically significant difference from the control group; #, *p* < 0.05; ##, *p* < 0.01; ###, *p* < 0.001; one-way ANOVA with Dunnett’s post hoc test. *, Statistically significant difference between B7 and SHB20 DEE; ***, *p* < 0.001. +, Statistically significant difference between treatment with and without DPF; +, *p* < 0.05; ++, *p* < 0.01; two-way ANOVA (biofuel type × DPF treatment) with Tukey’s post hoc test.

Dysregulation of colonic pro- and anti-inflammatory cytokines, including IL-6, TNF-α, IL-12, PGE2, and IL-10, may contribute to colonic tissue damage, colitis, and the development of colon cancer. As shown in Figure 4A, IL-6 concentration significantly increased in the SHB20 (+DPF) and SHB20 (-DPF) groups (+61% and +89%, respectively), compared to the control group. Compared with the corresponding B7 groups, IL-6 concentrations were markedly higher in SHB20 (+DPF) and SHB20 (-DPF) rats (+52% and +81%, respectively). DPF treatment significantly reduced IL-6 concentration by 17% in SHB20-exposed rats.

Similarly, TNF-α concentration (Figure 4B) was significantly increased in the SHB20 (+DPF) and SHB20 (-DPF) groups (+27% and +49%, respectively), compared to the control group, and was significantly higher than in the corresponding B7 groups (+21% and +33%, respectively). DPF treatment reduced TNFα concentration by 14% in SHB20-exposed rats.

As shown in Figure 4C, IL-12 concentration was significantly increased in all exposed groups [+20% in B7 (+DPF), +28% in B7 (-DPF), +96% in SHB20 (+DPF), and +112% in SHB20 (-DPF)], compared to the control group. IL-12 concentrations were markedly higher in SHB20 (+DPF) and SHB20 (-DPF) rats (+63% and +66%, respectively) than in the corresponding B7 groups, whereas DPF treatment had no effect.

PGE2 concentration (Figure 4D) was significantly increased in three exposed groups [+38% in B7 (-DPF), +19% in SHB20 (+DPF), and +49% in SHB20 (-DPF)], compared to the control group. No differences were observed between the B7 and SHB20 groups, whereas DPF treatment significantly decreased PGE2 concentration in the B7 (+DPF) and SHB20 (+DPF) groups (−21% and −25%, respectively) compared to the corresponding non-filtered groups.

In contrast, the concentration of the anti-inflammatory cytokine IL-10, as presented in Figure 4E, was significantly decreased in the SHB20 (+DPF) and SHB20 (-DPF) groups of rats (−66% and −65%, respectively), compared to the control group. When comparing B7 and SHB20 groups, markedly lower concentrations of IL-10 were observed in the SHB20 (+DPF) and SHB20 (-DPF) groups of rats (−63% and −65%, respectively), compared to the corresponding B7 (+DPF) and B7 (-DPF) groups of rats. Application of DPF treatment had no effect on IL-10 concentration.

### 2.5. Exposure to DEE from Combustion of the First-Generation B7 and the Second-Generation SHB20 Biofuels Without a DPF Is Accompanied by Subtle Histological Changes in the Colon Tissue of Rats

**Figure 5:**
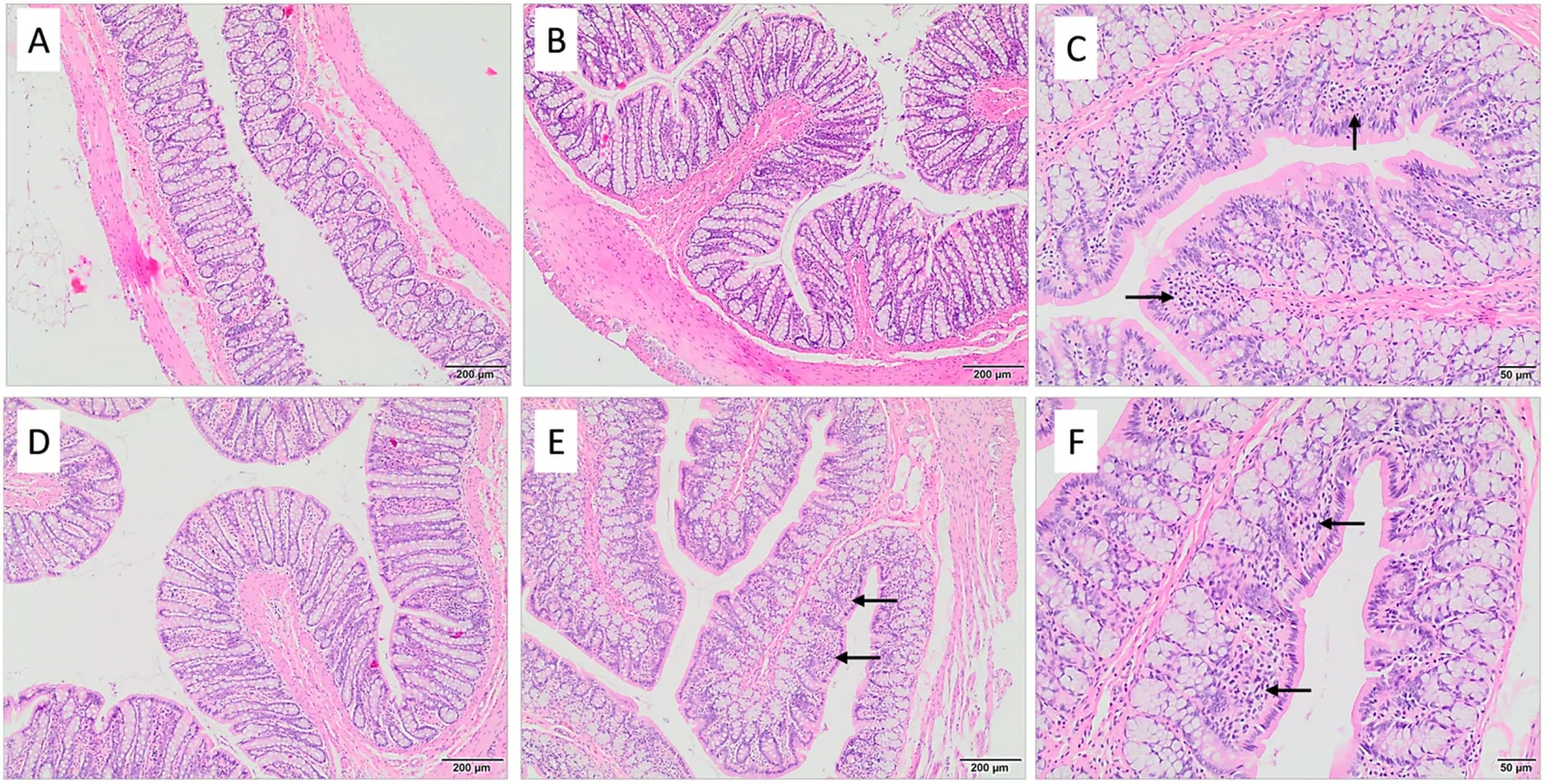
Representative microphotographs of haematoxylin–eosin-stained cross-sections of the colon tissue of rats: before DEE exposure (**A**), after exposure to DEE from B7 with DPF treatment for 7 days (**B**), after exposure to DEE from B7 without DPF treatment for 7 days (**C**), after exposure to DEE from SHB20 with DPF treatment for 7 days (**D**), after exposure to DEE from SHB20 without DPF treatment for 7 days ((**E**), lower magnification; (**F**), higher magnification). Images (**A**,**B**,**D**,**E**) were captured at 50× magnification; scale bars correspond to 200 µm. Images C and F were captured at 100× magnification; scale bars correspond to 50 µm. Arrows indicate foci of lymphocytic infiltration within the lamina propria. No colour coding is used; all sections were stained with haematoxylin and eosin.

Histological examination revealed subtle multifocal inflammatory lesions, lymphocytic infiltration, and mild shortening of the intestinal crypts in the colon tissue of rats exposed to B7 and SHB20 DEE without DPF treatment (Figure 5C,E,F). The lesions were of minimal severity. However, in the colon tissue of control rats and rats exposed to DEE from B7 and SHB20 biofuels with DPF treatment (Figure 5A,B,D), histological analysis revealed the typical architecture of the colon tissue.

## 3. Discussion

This study aimed to determine the influence of DEE derived from first (B7)- or second (SHB20)-generation biofuels, with and without DPF, on lipid peroxidation, antioxidant defence, inflammatory response, and subtle morphological alterations in rat colon tissues after 7-day whole-body inhalation. Exposure to both B7 and SHB20 emissions significantly increased TBARS and ISOPs in colon tissue, with stronger effects after SHB20 exposure. TBARS, including MDA, are aldehydes generated during ROS-induced decomposition of arachidonic acid and larger polyunsaturated fatty acids [23], whereas IsoPs are prostaglandin-like compounds formed by non-enzymatic ROS-mediated oxidation of arachidonic acid [24]. Since these compounds are considered the “gold standard” for assessing oxidative injury in vivo [25], our results show that both B7 and SHB20 DEE induced lipid peroxidation and oxidative stress in rat colon tissues, with SHB20 exerting the stronger effect. These findings complement our previous in vitro and in vivo studies showing that B7 and SHB20 emissions induced intracellular ROS production in A549 and BEAS-2B cells and increased lipid peroxidation in rat blood and testes [16,20,22]. However, data on DEE exposure and oxidant status in the gastrointestinal tract remain limited. Dybdahl et al. [26] reported increased *Ogg1* mRNA expression, a marker of oxidative stress, and DNA adduct formation in the colon of Big Blue rats after dietary exposure to DEE particles (0.2–80 mg/kg) for 21 days. Similarly, Risom et al. [27] showed that low-dose DEE particles (0.8 mg in feed) increased *Ogg1* mRNA expression, bulky DNA adducts, and strand breaks in the colon of Big Blue rats. In ApoE-/- mice, whole-body exposure to diluted DEE (~250 μg/m^3^) for 2 weeks increased MDA, 9- and 13-HODEs, and 5-HETE in the intestines and liver [28]. In 2019, the same group confirmed that ApoE-/- mice exposed to inhaled DEE (~250 μg/m^3^) for 16 weeks exhibited increased hepatic lipid peroxidation, as evidenced by elevated levels of IsoPs, HODEs, and 5-HETE [29]. More recently, Gupta et al. [30] showed that ApoE-/- mice exposed by whole-body inhalation to DEE (6 h/day, 5 days/week for 16 weeks) had altered caecal microbiome composition associated with hepatic peroxidised lipids (9- and 13-HODEs) and elevated plasma cholesterol and triglycerides.

Prolonged lipid peroxidation may exacerbate adverse cellular, tissue, and organ processes when ROS generation overwhelms antioxidant defence. This system includes enzymatic and non-enzymatic components, assessed either by TAS, which reflects total antioxidant capacity, or by individual components [31]. The present study evaluated both TAS and individual antioxidants. B7 and SHB20 emissions, with and without DPF, affected TAS in opposite directions: B7 DEE significantly increased TAS, whereas SHB20 DEE markedly decreased it. DPF treatment intensified these effects. Since elevated TAS generally indicates stronger defence against oxidative stress [32], B7 exposure probably triggered compensatory antioxidant mechanisms. In contrast, reduced TAS after SHB20 exposure suggests an imbalance between ROS generation and antioxidant capacity, potentially impairing colon tissue function. These opposing TAS effects are most likely related to emission composition. Although comparative analysis showed no marked differences in CO, CO_2_, NOx, and PM concentrations, B7 DEE had ~1.5-fold higher HC concentration than SHB20 DEE, whereas SHB20 DEE showed distinct qualitative particulate composition. Detailed HC analysis showed similar PAH types, but total PAH content was ~2.4-fold higher in B7 DEE than in SHB20 DEE. DPF reduced PAHs by ~1000-fold in B7 DEE and ~250-fold in SHB20 DEE, ultimately giving ~2-fold higher PAH content in SHB20 DEE than B7 DEE. Since PAHs can cause oxidative damage through excessive ROS production [33], the higher PAH content in SHB20 DEE likely contributed to its greater toxicity, particularly in the absence of DPF. Another factor was the higher concentration of redox-active transition metals (iron, zinc, copper) and the exclusive presence of silver in particles from SHB20 biofuel combustion. Iron, copper, and zinc induce oxidative stress by catalysing the Fenton and Haber–Weiss reactions, generating ROS [34], whereas silver, especially in nanoparticle or ionic forms, may disrupt the mitochondrial respiratory chain or act via Fenton-like reactions [35]. Higher SHB20 toxicity may also be related to greater numbers of ultrafine particles in the 10–90 nm range (85% vs. 55%), as smaller particles induce higher ROS levels and oxidative potential [36]. The literature on TAS and DEE exposure is inconclusive. Our previous study showed increased TAS in rat testes after B7 and SHB20 DEE exposure (2% DEE, 6 h/day, 5 days/week, 4 weeks) [16]. Conversely, Nemmar et al. [37] reported decreased TAS in erythrocytes of mice exposed intratracheally to DEE particles (30 mg/mouse for 4 or 18 h), with no effect on human erythrocytes. Different DEE systems, fuels, and models probably explain these discrepancies.

Apart from TAS, we evaluated key antioxidant enzymes: SOD, GPx, and GR, which form the first line of defence against oxidative stress [31]. SOD accelerates the dismutation of the superoxide anion (O_2_•−) to H_2_O_2_ and O_2_ [38]. GPx reduces peroxides, including organic hydrogen peroxides, to water or the corresponding alcohols, protecting cell membranes from peroxide damage [39]. GR catalyses the reduction of GSSG to GSH in the presence of NADPH [40]. In our study, SOD and GPx activities in the colon tissue increased significantly after B7 and SHB20 DEE exposure, with and without DPF. Higher SOD and GPx activities were observed after SHB20 than after B7 exposure, particularly without DPF. This likely reflects a compensatory physiological response to ROS overproduction, although elevated SOD and GPx may imbalance cellular antioxidant defence by accelerating H_2_O_2_ generation. Data on SOD and GPx in DEE-exposed animals are contradictory. Morin et al. [41] reported that exposure of rats to diluted DEE (2% standard diesel fuel) increased SOD, GPx, and catalase activities in lung tissue. Cattani-Cavalieri et al. [42] showed that chronic intranasal exposure of mice to DEE particles (30 µg/mice for 60 days) increased lung GPx but did not change SOD. However, Nemmar et al. [37] reported unchanged SOD activity 4 h after acute exposure to DEE particles (30 µg/mouse), but a significant decrease after 18 h. Moreover, Gao et al. [43] reported decreased SOD in serum, aorta, and liver after DEE exposure (4 h/day for 5 days), but increased GSH-Px in these tissues. These discrepancies likely reflect differences in fuel, operating conditions, exhaust treatment, exhaust composition, exposure duration, organism, and tissue.

Another finding was differential GR regulation. Increased GR activity in colon tissues from rats exposed to B7 DEE without DPF was accompanied by elevated GSH, suggesting enhanced GSH regeneration. In contrast, SHB20 DEE decreased GR activity, with or without DPF. Since GR is essential to the glutathione system, increased GR activity may indicate overcoming oxidative stress by regenerating GSH from GSSG, whereas decreased GR activity may promote oxidative stress by reducing GSH availability [40]. Accordingly, B7 DEE significantly increased GSH concentration, whereas SHB20 markedly decreased it and dramatically increased GSSG. DPF treatment intensified the reduction in GSSG in colon tissues from SHB20-exposed rats. Both B7 and SHB20 DEEs decreased the GSH/GSSG ratio, but the lower ratio occurred after SHB20 exposure. Since reduced intracellular GSH and/or GSH/GSSG ratio indicate(s) cellular redox imbalance [44], SHB20 DEE appears to accelerate GSH oxidation to GSSG more strongly than B7 DEE.

The factorial analysis showed a significant biofuel × DPF interaction for eight of the 14 biomarkers, with partial eta-squared ranging from 0.17 to 0.92 (Supplementary Table S2). For TAS and glutathione reductase the interaction was qualitative rather than merely quantitative, with particle filtration acting in opposite directions in the two blends: in B7-exposed animals the filter raised TAS and lowered GR activity, whereas after SHB20 exposure it lowered TAS and raised GR activity. For GSH and the GSH/GSSG ratio the filter had a significant effect only in the B7-exposed animals, lowering GSH and raising the GSH/GSSG ratio, with no significant effect on the SHB20-exposed animals. The effect of the filter on the colon therefore cannot be described independently of the fuel blend, and for these endpoints the main effect of filtration is not interpretable in isolation.

Redox imbalance plays a pivotal role in inflammatory signalling [45]. We therefore assessed IL-6, TNFα, IL-12, PGE-2, and IL-10. IL-6 regulates inflammation, immune responses, and multiple physiological processes [46]. TNF-α strongly activates cytokine production and inflammatory and immune responses [47]. IL-12 induces IFN-γ production and participates in adaptive and innate immunity, including inflammation [48]. PGE_2_ is a bioactive lipid from the oxidation of 20-carbon essential fatty acids and mediates inflammatory effects [49]. In contrast, IL-10 is anti-inflammatory and limits uncontrolled immune responses [50]. SHB20-derived DEE significantly increased IL-6, TNFα, IL-12, and PGE_2_ in colon tissues, while B7 DEE induced only modest increases in IL-12 and PGE_2_. IL-10 decreased significantly only after SHB20 exposure. These changes indicate a significant pro-inflammatory effect of SHB20 DEE and a weaker effect of B7 DEE. These results corroborate our previous findings that SHB20-derived DEE particles more strongly induced IL-6 release in BEAS-2B airway epithelial cells than B7-derived particles [51]. DEE is a well-established inflammatory inducer, especially in the respiratory system [52], but direct evidence for gastrointestinal inflammation remains scarce. Bosch et al. [53] reported that oral exposure of mice to DEE particles (12 μg/day, 5 days/week) for 10 months shifted intestinal macrophage subpopulations toward an inflammatory milieu by reducing CCR2-negative anti-inflammatory/resident macrophages. The transcriptional response in colonic macrophages included an inflammatory response and interferon activation. Phillippi et al. [54] showed that oropharyngeal aspiration of DEE particles (35 μg/day, 2×/week) for 30 days altered small-intestinal microbial profiles and increased systemic pro-inflammatory cytokines, including IL-1α, IL-3, IL-13, IL-15, G-CSF, LIF, MIP-2, and TNF-α. Conversely, the same study reported decreased expression of TNF-α and IL-10 in the duodenum, jejunum, and ileum [55]. Emerging evidence links oxidative stress and inflammatory signalling with gut microbiota composition. DEE particles and associated organic compounds may induce dysbiosis, thereby further amplifying intestinal inflammation through increased epithelial permeability, activation of pattern-recognition receptors, and altered short-chain fatty acid production [56].

Sustained release of pro-inflammatory cytokines may cause morphological changes in colon tissue [57]. In the present study, we observed subtle multifocal inflammatory lesions, lymphocytic infiltration, and subjective shortening of intestinal crypts in rats exposed to B7 and SHB20 DEEs without DPF. These lesions were of minimal severity and were recorded at a single timepoint, immediately after the end of the exposure period. Because the design included neither a post-exposure recovery period nor any later assessment, whether such changes resolve, persist or progress cannot be determined from the present data. Studies on airborne pollutants link PM2.5 and PM10 exposure with colon morphological changes, including crypt shortening, epithelial cell loss, goblet cell changes, mucus layer thinning, villi damage, and mononuclear and/or neutrophilic infiltration [58], but DEE effects on colon histopathology remain largely unknown. Liu et al. [59] reported that exposure of C57BL/6J mice to DEE particles (5 mg/kg bw every 2 days) for 14 days by intratracheal instillation deteriorated the gastrointestinal tract and altered gut microbiota structure and composition, but did not significantly affect intestinal villi length, goblet cells, or villus epithelial cells in the colon. Conversely, Phillippi et al. [55] observed increased goblet cell production in the intestines of C57Bl/6 male mice exposed to DEE particles (35 μg/day, 2×/week) by oropharyngeal aspiration for 30 days.

A clear limitation of this study was the sequential processing of the different exposure series and control group. Although using two parallel chambers simultaneously provided an ideal, direct control for the DPF filtration effect within each individual DEE exposure, the sequential block design across different biofuels and controls could not fully eliminate potential run-specific confounding effects. Therefore, future studies utilising multi-chamber systems to run all experimental arms and controls completely in parallel are warranted to fully corroborate these sequential comparisons.

Another limitation of the study is that the route by which inhaled emissions reach the colon cannot be resolved. Whole-body exposure permits pulmonary deposition followed by systemic distribution of soluble and particle-associated constituents, direct gastrointestinal delivery through mucociliary clearance and swallowing of deposited particles, and ingestion associated with grooming of contaminated fur, and these routes cannot be distinguished with the endpoints measured here. The changes described above should therefore be interpreted as colonic responses to whole-body emission exposure, and not as a demonstrated direct effect of inhaled emissions on colon tissues.

Several methodological considerations temper the mechanistic interpretation of the measured endpoints. The TBARS assay is relatively non-specific and reports a range of thiobarbituric acid-reactive aldehydes rather than malondialdehyde alone; TAS is an integrated measure of the reducing capacity of the tissue homogenate and not a direct measure of antioxidant protection; and cytokine concentrations determined in whole-colon homogenates identify neither the cell populations that produced them nor whether the cellular composition of the tissue changed during exposure. The histological evaluation was descriptive, based on representative sections without a predefined semi-quantitative scoring system, so the statement that exposure altered colon architecture is to be read as a qualitative observation. Accordingly, the present findings are described as oxidative-stress-associated, redox-related and inflammatory-associated alterations rather than as proof of a defined mechanism.

The mass of particles collected downstream of the diesel particulate filter was insufficient for physicochemical analysis, so the particle composition of the filtered emissions was not determined. Statements relating the colonic effects of filtered emissions to the removal of polycyclic aromatic hydrocarbons, redox-active transition metals or ultrafine particles are therefore inferential. A particulate filter may also modify the gaseous and particle-associated composition of the exhaust beyond particle mass, and the unexpectedly higher hydrocarbon concentrations measured downstream of the filter in both blends indicate that such changes occurred; claims of a direct protective action of the filter are consequently formulated with caution.

The relevance of this model to human exposure requires careful consideration. Rats were exposed whole-body in a closed chamber to 2% diluted exhaust for seven consecutive days, which produced particulate matter concentrations roughly two orders of magnitude above typical urban and roadside ambient levels and, expressed as particulate mass, above the binding European Union occupational exposure limit for diesel engine exhaust emissions of 0.05 mg/m^3^ elemental carbon. The model therefore approximates a short, high-intensity, near-source or occupational-type exposure rather than chronic exposure of the general population, and the obligate nasal breathing, higher ventilation rate per body mass and different gastrointestinal physiology of the rat further limit direct quantitative extrapolation. The present findings should accordingly be read as evidence that biodiesel exhaust can reach and affect the colon under controlled high-level exposure, and not as an estimate of risk at ambient concentrations in humans.

## 4. Materials and Methods

### 4.1. Animals

Healthy adult male Fischer 344 rats (*n* = 35) were obtained from Charles River Laboratories, Inc. (Schulzfeld, Germany). The rats were allowed to acclimatise to animal-house conditions at the Institute of Veterinary Medicine for 1 week (22 ± 1 °C, 50 ± 5% relative humidity, 12 h light–dark cycle). The rats had free access to water and food ad libitum and were fed with AIN-93M maintenance pellets for laboratory rats (ZooLab, Sędziszów, Poland). The use of animals and all procedures were approved by the III Local Animal Care and Use Committee, Warsaw, Poland (Resolution # 4/2014, 22 January 2014), in accordance with Polish legal regulations and the 3R rule (Replacement, Reduction, and Refinement).

### 4.2. Exposure of Animals to DEE

After the adaptation period, rats were randomly assigned to 5 groups: 4 experimental groups and 1 control group, with 7 animals per group; all 35 animals completed the exposure protocol and none was excluded. The exposure of rats to DEE was previously described by our group [8,9,12,19,20]. Briefly, the DEE was generated from a Fiat Panda 1.3 JTD (2014) (Fiat, Turin, Italy) with a Euro 5 engine (Common Rail 3rd-generation injection system, 1248 cm^3^, max. power of 75 bhp, max. torque of 190 Nm). The engine was run under controlled conditions on a chassis dynamometer (Schenck Komeg EMDY 48) at static speed and load, 1340 rpm and 45.7%, respectively, corresponding to a constant vehicle speed of 43.75 km/h. The animals from experimental groups were exposed in whole-body inhalation chambers to the DEE from two biodiesel blends [B7, the 1st-generation biofuel containing 7% (*v*/*v*) FAME, or SHB20—the 2nd-generation biofuel containing 7% (*v*/*v*) FAME and 13% (*v*/*v*) HVO] diluted to 2% with air. Rat exposure was performed separately for each biodiesel blend type. For each of the four exposure conditions a single whole-body inhalation chamber was used, with seven rats per chamber, so that the chamber, and not the individual animal, constitutes the unit of randomisation for the emission treatment; the consequences of this design constraint are addressed in the Discussion Section. The engine generated two exhaust gas streams, which were supplied to the inhalation chambers. One stream was collected after exhaust filtration through a diesel particle filter (+DPF), and another was collected before the particle filter (-DPF). The measured concentrations of particulate matter and gaseous components in the chambers are given in Supplementary Table S1. Particulate matter amounted to 2.19 ± 0.13 and 2.13 ± 0.13 mg/m^3^ in the B7 (-DPF) and SHB20 (-DPF) chambers and to 0.17 ± 0.01 and 0.19 ± 0.01 mg/m^3^ in the B7 (+DPF) and SHB20 (+DPF) chambers, with NOx of 41.3 ± 2.50 and 41.9 ± 2.55 g/m^3^ without DPF and 38.0 ± 2.00 and 38.4 ± 2.00 g/m^3^ with DPF, and CO of 2.60 ± 0.16 and 2.71 ± 0.17 g/m^3^ without DPF and below the limit of quantification with DPF. Exhaust dilution was verified continuously from the chamber carbon dioxide concentration (set value of 3.60 g/m^3^, measured at 3.62–3.63 g/m^3^), and the exhaust dilution ratio was 2.14–2.26%. The other chamber conditions were as follows: internal carbon dioxide concentration was 2000 ± 50 ppm, internal air exchange rate was 25 ± 1/h, relative air humidity was 50 ± 5% and air temperature was 23 ± 1 °C. The inhalation procedure was performed for seven consecutive days (7 h/day), giving seven exposure sessions and 42 h of total exposure per animal. Control rats were housed within the same inhalation chambers during a dedicated sequential run, receiving only filtered air. Throughout the experiment they were maintained under standard animal-house conditions (22 ± 1 °C, 50 ± 5% relative humidity, 12 h light–dark cycle, free access to water and the same AIN-93M diet) and breathed clean, charcoal-filtered room air. This was a deliberate choice. The reference condition for the present study was intended to represent the undisturbed physiological state of the gastrointestinal tract, and gastrointestinal function is particularly responsive to the confinement, restricted locomotion, continuous forced airflow and elevated carbon dioxide concentration that whole-body chamber housing necessarily entails; intestinal motility, feed and water intake, and the composition and transit of the intestinal content all respond to these factors. In our view, housing the controls under standard conditions therefore provides a fully faithful representation of normal physiological functioning of the rat digestive tract, which chamber housing would not have preserved. The consequence for interpretation, namely that the contrast between exposed and control animals also contains the contribution of chamber housing itself, whereas the comparisons among the four chamber-housed exposure groups do not, is addressed explicitly in Section 3. Upon the completion of the inhalation procedure, rats were anaesthetised with isoflurane (Aerrane Isofluranum, Baxter, Deerfield, IL, USA) and bled out by heart puncture. Next, colon samples from each rat were dissected, washed with ice-cold PBS, immediately frozen in liquid nitrogen, and stored at −80 °C for biochemical analyses, or fixed in 10% formalin for histopathological evaluation. The 7-day, 6 h/day regimen corresponds to the short-term exposure arm of the FuelHealth project [8,9,12,20] and was chosen to capture early biochemical and morphological responses in the colon. It is shorter than the 28-day subacute inhalation protocol of OECD Test Guideline 412, and the present data therefore characterise acute-to-early-subacute effects and do not support inference about medium- or long-term outcomes. With respect to dose, the particulate matter concentration used without DPF (approximately 2.1–2.2 mg/m^3^) exceeds typical urban and roadside ambient levels, at which PM2.5 generally remains below 0.03 mg/m^3^, and, expressed as particulate mass, lies above the binding European Union occupational exposure limit for diesel engine exhaust emissions of 0.05 mg/m^3^ measured as elemental carbon. The exposure level thus represents a high-intensity, near-source or occupational-type scenario selected to elicit measurable responses within a short exposure period, rather than average ambient human exposure.

### 4.3. Oxidative Stress and Antioxidant Defence System Parameters in the Colon Tissue

Fragments of colon tissue were homogenised in cold phosphate buffer (PBS) (50 mM potassium phosphate, pH 7.5, with 1 mM EDTA) at a tissue-to-buffer ratio of 1:10 using the Bio-Gen PRO200 homogeniser. The obtained homogenates were centrifuged (10,000× *g*, 15 min, 4 °C) and the supernatants were aliquoted for subsequent assays. The level of lipid peroxidation was determined by measuring the concentration of thiobarbituric acid-reactive species (TBARS), expressed as malondialdehyde (MDA) equivalents and secondary products of lipid peroxidation [60]. TBARS concentrations were calculated from a standard curve prepared using 1,1,3,3-tetramethoxypropane, which yields MDA upon hydrolysis during the assay. Total antioxidant status (TAS), activity of superoxide dismutase (SOD), activity of glutathione peroxidase (GPx) and activity of reductase (GR) were determined spectrophotometrically using Randox assay kits (Randox Laboratories Ltd., Crumlin, Country Antrim, UK) (cat no: NX2332, SD125, RS505, GR2368, respectively), according to the manufacturer’s protocols. The activities of antioxidative enzymes were expressed in universal units per gram of tissue (U/g). Concentrations of reduced (GSH) and oxidised (GSSG) forms of glutathione were determined by high-performance liquid chromatography (HPLC) with a coulometric electrochemical detector (ESA, Chelmsford, MA, USA) according to procedures previously described by us [61]. Briefly, we employed a 4-channel electrochemical array for the simultaneous detection of GSH and GSSG. The mobile phase for isocratic elution of sulfhydryls was composed of 25 mM monobasic sodium phosphate, 0.5 mM 1-octanesulfonic acid (ion-pairing agent), and 2.5% acetonitrile, pH 2.7. The pH for the mobile phase was adjusted with 85% phosphoric acid. A flow rate of 1 mL min−1 was used with a C18 column (5 μM column, 4.6 × 250 mm). Acetonitrile was the key component in modulating the retention times of GSH and GSSG. With 2.5% acetonitrile in the mobile phase, the retention times for GSH were generally 5 min, and for GSSG, 20 min. Concentration of 8-isoprostane was determined using an ELISA Kit (cat. no: 516351, Cayman Chemical, Ann Arbour, MI, USA) according to the manufacturer’s instructions.

### 4.4. Immunoenzymatic Determination of Concentrations of Selected Cytokines and Prostaglandin E2 in the Colon Tissue

Concentrations of pro- and anti-inflammatory cytokines, including interleukin 6 (IL-6) (cat. no: EIA-4640), interleukin 12 (IL-12) (cat. no: EIA-4843), interleukin 10 (IL-10) (cat. no: EIA-4699) and tumour necrosis factor alpha (TNF-α) (cat. no: EIA-4641) were determined by a competitive specific enzyme immunoassay (ELISA) following manufacturer’s instructions (DRG Instruments GmbH, Marburg, Germany). Concentration of prostaglandin E2 (PGE2) was measured spectrophotometrically using a monoclonal ELISA Kit (cat. no: 363-24-6, Cayman Chemical, Ann Arbor, MI, USA).

### 4.5. Histopathological Evaluation of the Colon Tissue

Colon tissue samples were fixed in 10% formalin and dehydrated by successive immersion in a gradient of ethanol solutions. The samples were then rinsed with xylene, embedded in paraffin blocks, and cut into 6 μm sections. Colon tissue sections were stained with haematoxylin and eosin (H&E) for evaluation under a light microscope (Motic BA400, Olympus Corporation, Tokyo, Japan) by a veterinary pathologist. The assessment was descriptive and was not based on a predefined semi-quantitative lesion-scoring system; all sections were evaluated by the same observer. Accordingly, the histopathological findings are reported qualitatively, are presented as representative images, and were not subjected to statistical analysis.

### 4.6. Statistical Analysis

Statistical analyses were conducted using Statistica software version 13.3 (Data Analysis Software System, 2014). All data were analysed using a two-way analysis of variance (ANOVA), followed by Tukey’s post hoc test, with biofuel type (B7, SHB20) and DPF treatment (+DPF, -DPF) as factors and their interaction (biofuel × DPF) included in the model. This model comprised the 4 experimental groups, which constitute a 2 × 2 factorial design, and a sigma-restricted parameterisation with effective hypothesis decomposition was used. The control group was not included in this model, as it was not assigned to either factor. Where the interaction was significant, the main effects were not interpreted separately, and the effect of DPF treatment was evaluated within each biofuel using Tukey’s post hoc test applied to the means of the 4 experimental groups. Comparisons between the experimental groups and the control group were defined a priori as planned contrasts against a single reference group and were performed using one-way ANOVA followed by post hoc Dunnett’s test. Normal distribution and equality of variances were tested for all data. Data that did not meet these assumptions were log-transformed before analysis (TBARS and IL-10), and untransformed values are presented in the figures. Statistical significance was set at *p* < 0.05. All the results were presented as mean ± SD (Standard Deviation). F values with the corresponding degrees of freedom, exact *p* values and partial eta-squared (η^2^p) as a measure of effect size are given for both main effects and for the interaction in Supplementary Table S2, and for the one-way model in Supplementary Table S3. The 14 biomarkers were selected a priori as a mechanistically related panel addressing the induction of oxidative stress and of an inflammatory response and were not treated as independent confirmatory tests; the family-wise error rate within each biomarker is controlled by Dunnett’s and Tukey’s tests, and no correction was applied across biomarkers. Applying the Benjamini–Hochberg procedure across the 14 biomarkers, separately for each term of the model, changed the significance of only 2 of the 42 tests. The figures were performed in SigmaPlot version 14.0 for Windows (Systat Software, Inc., San Jose, CA, USA).

## 5. Conclusions

In conclusion, this study provides evidence that even short-term (7-day) whole-body exposure of rats to DEE, derived from first (B7)- or second (SHB20)-generation biofuels with and without DPF, induces oxidative-stress-associated and redox-related changes, alters antioxidant defence mechanisms, promotes inflammatory-associated responses, and is accompanied by subtle morphological alterations in rat colon tissues. The application of DPF markedly decreased the concentration of particulate matter and the total content of PAHs in both B7 and SHB20 exhaust emissions, but its effects on the colonic endpoints were partial and endpoint-dependent: filtration reduced or altered some markers, left others unchanged, and in several cases acted in opposite directions for the two blends, so that a reduction in particulate matter or PAH content did not translate into a normalisation of the biological response. Effects of SHB20 DEE were greater than those observed for B7 DEE across the endpoints examined. Because the two blends differ simultaneously in fuel type, total renewable-fuel fraction and chemical composition, these differences are provisionally ascribed to blend-specific properties of the emissions, including exhaust composition, the total content of PAHs, the concentration of redox-active transition metals and the content of particles in the ultrafine-size range, rather than to biodiesel generation as such. This attribution is consistent with the chemical characterisation of the emissions but was not tested directly. Whether the alterations observed after seven days of high-level exposure translate into a clinically meaningful risk of inflammatory colonic disease cannot be determined from the present design, and longer exposures combined with disease-prone models and direct assessment of barrier function and microbiota are required to address this question. Because all endpoints were measured at a single timepoint at the end of exposure, the present design cannot establish whether the responses observed are transient or persistent, and studies including a post-exposure recovery period are needed to resolve this.

These findings should be regarded as exploratory, given the single inhalation chamber per exposure condition, the group size of seven animals and the number of endpoints examined. The results are presented as a coherent pattern of oxidative-stress-associated and inflammatory-associated alterations, and the blend- and filter-specific effects described above require confirmation in an independent experiment.

The present study has certain limitations, including the relatively short exposure duration and reliance on a single animal model. Furthermore, gut barrier integrity and microbiome composition were not assessed, because colon samples were collected and preserved according to a protocol designed for biochemical and histological endpoints and no material suitable for permeability measurement or 16S rRNA sequencing was retained. Their contribution can therefore neither be quantified nor excluded, and since an altered epithelial barrier or microbiota could amplify or attenuate every response reported here, the mechanistic sequence linking inhalation exposure to colonic inflammation remains open. Moreover, the histopathological examination comprised only a descriptive assessment of the morphological changes in the colon mucosa, so quantitative changes could not be determined unambiguously or subjected to statistical analysis. Body weight and feed intake were not analysed as endpoints in the present study, so the biomarker concentrations, which are expressed per gram of tissue, cannot be related to possible changes in the general condition of the animals during exposure. Therefore, future research is needed to integrate multi-omics approaches, longer exposure scenarios, post-exposure recovery periods, and disease-prone models to better understand the long-term gastrointestinal consequences of biofuel-derived DEE.

## Data Availability

The data presented in this study are available from the corresponding author upon reasonable request.

## Supplementary Materials

The following supporting information can be downloaded at: https://www.mdpi.com/article/10.3390/ijms27219724/s1.
