# Supplementary material for: Colon Effects of 7-Day Whole-Body Inhalation Exposure of Fischer 344 Rats to Emissions from First- and Second-Generation Biodiesel Fuels with and Without a Particle Filter
Source: Int J Mol Sci. 2026 Oct 29;27(21):9724. doi: 10.3390/ijms27219724 (PMC13762620; doi:10.3390/ijms27219724)
Supplement: Supplementary file 1 [file ijms-27-09724-s001.zip › Supplementary_Materials_10.12.pdf]

Supplementary Materials

Supplementary Table S1. The estimated concentration of selected substances in the test chambers. LoQa – below the limit of quantification (0.01% vol.). CO_2 - carbon dioxide, CO - carbon monoxide, HC - unburned hydrocarbons, NOx - nitrogen oxides, PM - particle matter (Dziendzikowska et al. 2018).

Component	diesel B7 blend				diesel SHB20 blend			
	- DPF		+ DPF		- DPF		+ DPF	
	Mean	SD	Mean	SD	Mean	SD	Mean	SD
CO_2 [g/m^3] (set value)	3.6	-	3.60	-	3.60	-	3.60	-
Exhaust dilution ratio [%]	2.17	0.08	2.14	0.003	2.26	0.08	2.19	0.03
CO_2 [g/m^3]	3.63	0.22	3.62	0.19	3.63	0.22	3.62	0.19
CO [g/m^3]	2.60	0.16	LoQ	LoQ	2.71	0.17	LoQ	LoQ
HC [g/m^3]	0.79	0.05	0.90	0.05	0.55	0.03	1.07	0.06
NOx [g/m^3]	41.3	2.50	38.00	2.00	41.90	2.55	38.4	2.00
PM [mg/m^3]	2.19	0.13	0.17	0.01	2.13	0.13	0.19	0.01

Dziendzikowska K, Gajewska M, Wilczak J, Mruk R, Oczkowski M, Żyła E, Królikowski T, Stachoń M, Øvrevik J, Myhre O, Kruszewski M, Wojewódzka M, Lankoff A, Gromadzka-Ostrowska J, 2018. The effects of 1st and 2nd generation biodiesel exhaust exposure on hematological and biochemical blood indices of Fisher344 male rats – The FuelHealth project. Environ Toxicol Pharmacol 63. https://doi.org/10.1016/j.etap.2018.08.001

Supplementary Table S2. Results of two-way analysis of variance for the four experimental groups, which constitute a 2 × 2 factorial design with biofuel type (B7, SHB20) and DPF treatment (+DPF, -DPF) as factors. TBARS and IL-10 were analysed after log transformation. Each F value has one degree of freedom in the numerator. p values below 0.001 are reported as <0.001. η^2p, partial eta squared, calculated as SSeffect / (SSeffect + SSerror). TBARS, thiobarbituric acid reactive substances; TAS, total antioxidant status; GR, glutathione reductase; GPx, glutathione peroxidase; SOD, superoxide dismutase; GSH, reduced glutathione; GSSG, glutathione disulfide; IL-6, interleukin 6; TNF-α, tumour necrosis factor α; IL-12, interleukin 12; PGE2, prostaglandin E2; IL-10, interleukin 10.

**Biomarker**	**df**	**Biofuel type**			**DPF treatment**			**Biofuel × DPF**		
		**F**	**p**	**η^2p**	**F**	**p**	**η^2p**	**F**	**p**	**η^2p**
TBARS	1, 23	24.71	<0.001	0.518	83.74	<0.001	0.785	0.39	0.538	0.017
8-isoprostanes	1, 24	201.96	<0.001	0.894	7.43	0.012	0.236	0.22	0.643	0.009
TAS	1, 21	681.92	<0.001	0.970	2.06	0.166	0.089	242.87	<0.001	0.920
GR	1, 22	562.70	<0.001	0.962	4.39	0.048	0.166	147.28	<0.001	0.870
GPx	1, 24	47.04	<0.001	0.662	10.65	0.003	0.307	7.06	0.014	0.227
SOD	1, 24	121.31	<0.001	0.835	61.49	<0.001	0.719	22.09	<0.001	0.479
GSH	1, 24	372.99	<0.001	0.940	47.65	<0.001	0.665	69.07	<0.001	0.742
GSSG	1, 23	26.98	<0.001	0.540	3.88	0.061	0.144	4.65	0.042	0.168
GSH/GSSG	1, 23	229.64	<0.001	0.909	5.28	0.031	0.187	34.24	<0.001	0.598
IL-6	1, 23	237.77	<0.001	0.912	8.09	0.009	0.260	10.89	0.003	0.321
TNF-α	1, 23	45.14	<0.001	0.662	9.25	0.006	0.287	3.17	0.088	0.121
IL-12	1, 24	292.34	<0.001	0.924	6.68	0.016	0.218	0.78	0.386	0.031
PGE2	1, 23	5.13	0.033	0.182	52.76	<0.001	0.696	0.51	0.483	0.022
IL-10	1, 20	484.90	<0.001	0.960	3.30	0.086	0.142	1.00	0.340	0.048

Supplementary Table S3. Results of one-way analysis of variance with group as a single five-level factor (control and the four experimental groups), used as the omnibus test preceding the planned contrasts of each experimental group against the control. TBARS and IL-10 were analysed after log transformation. All p values were below the display precision of the software and are reported as <0.001. η^2p, partial eta squared. TBARS, thiobarbituric acid reactive substances; TAS, total antioxidant status; GR, glutathione reductase; GPx, glutathione peroxidase; SOD, superoxide dismutase; GSH, reduced glutathione; GSSG, glutathione disulfide; IL-6, interleukin 6; TNF-α, tumour necrosis factor α; IL-12, interleukin 12; PGE2, prostaglandin E2; IL-10, interleukin 10.

**Biomarker**	**df**	**F**	**p**	**η^2p**
TBARS	4, 29	69.11	<0.001	0.905
8-isoprostanes	4, 30	87.18	<0.001	0.921
TAS	4, 27	183.73	<0.001	0.965
GR	4, 27	167.73	<0.001	0.961
GPx	4, 30	39.76	<0.001	0.841
SOD	4, 30	118.86	<0.001	0.941
GSH	4, 27	114.87	<0.001	0.944
GSSG	4, 29	19.38	<0.001	0.728
GSH/GSSG	4, 28	66.43	<0.001	0.905
IL-6	4, 29	80.01	<0.001	0.917
TNF-α	4, 29	23.43	<0.001	0.764
IL-12	4, 30	129.96	<0.001	0.945
PGE2	4, 28	27.24	<0.001	0.796
IL-10	4, 25	164.11	<0.001	0.963

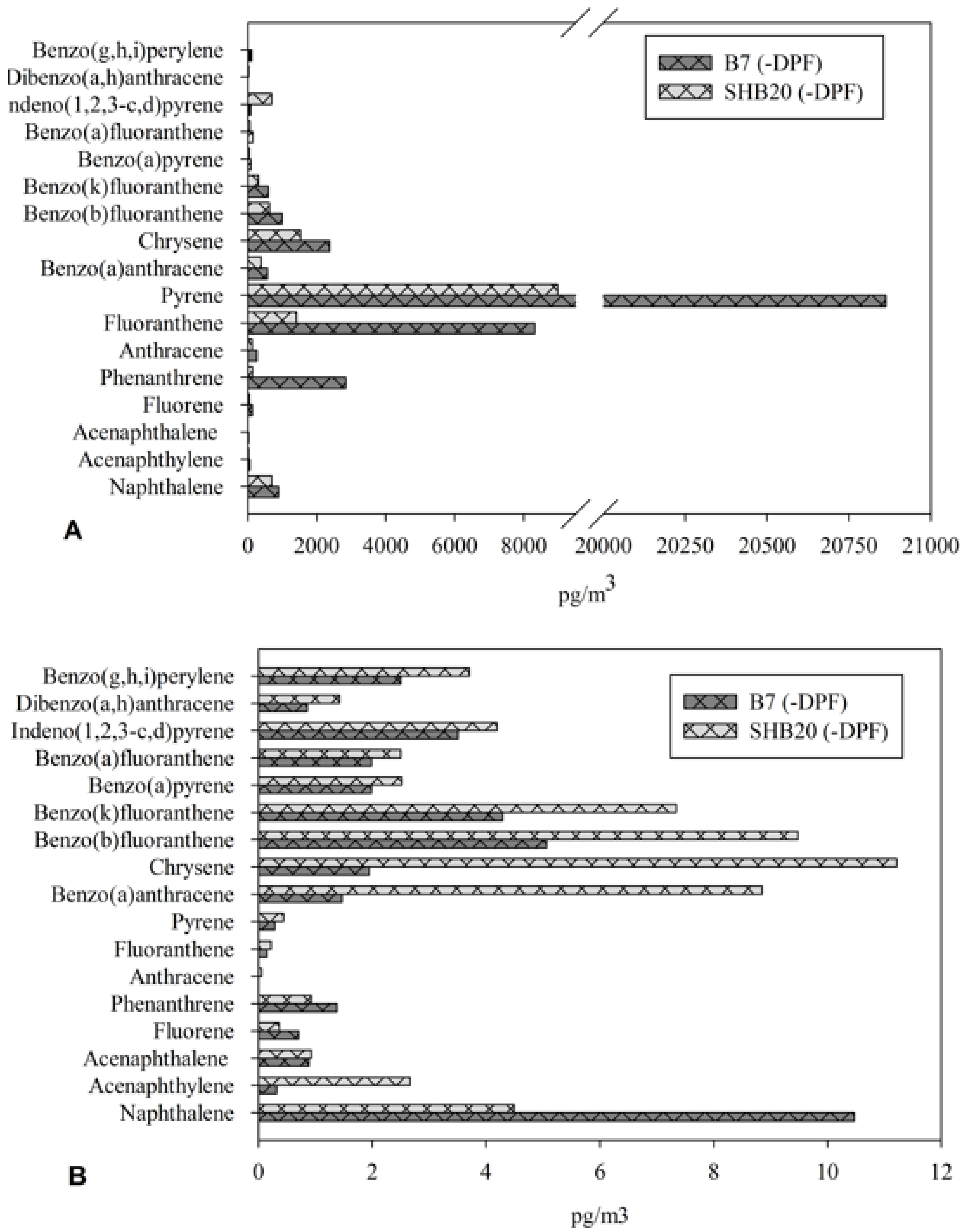

Supplementary Figure S1. Characteristics of air composition inside the inhalation chambers. (A) The estimated concentration (pg/m3) of selected aromatic hydrocarbons in the test chambers containing the B7 and the SHB20 exhaust emissions without DPF, (B) the estimated concentration (pg/m3) of selected aromatic hydrocarbons in the test chambers containing the B7 and the SHB20 exhaust emissions with DPF.

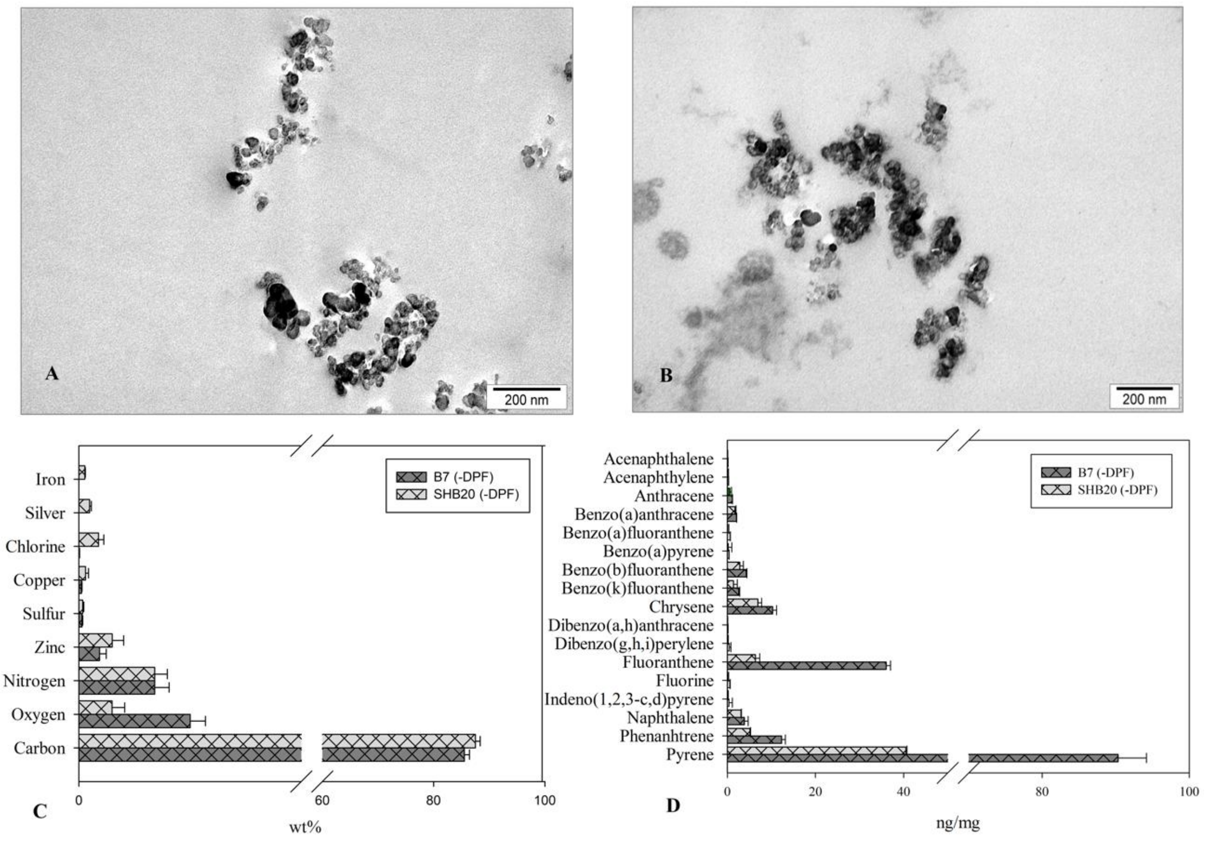

Supplementary Figure S2. Characteristics of PM inside the inhalation chambers, generated from combustion of B7 and SHB20 biofuels without a DPF filter. TEM images of particles generated from the combustion of B7 (A) and SHB20 (B) biofuels. A scale bar corresponds to 200 nm. (C) Elemental composition (wt%) of particles generated from the combustion of B7 and SHB20 biofuels. Data are presented as mean ± SD from 3 independent measurements (samples) by SEM-EDS, (D) Concentration (ng/mg) of selected aromatic hydrocarbons in the organic extracts from particles generated from combustion of B7 and SHB20 biofuels. Data are presented as mean ± SD from 3 independent measurements (samples) by GC-MS.
